# Hinokitiol Exerts Anticancer Activity through Downregulation of MMPs 9/2 and Enhancement of Catalase and SOD Enzymes: *In Vivo* Augmentation of Lung Histoarchitecture

**DOI:** 10.3390/molecules201017720

**Published:** 2015-09-25

**Authors:** Chien-Hsun Huang, Thanasekaran Jayakumar, Chao-Chien Chang, Tsorng-Harn Fong, Shing-Hwa Lu, Philip Aloysius Thomas, Cheuk-Sing Choy, Joen-Rong Sheu

**Affiliations:** 1 Graduate Institute of Medical Sciences, Taipei Medical University, Taipei 110, Taiwan; E-Mail: DAR87@tpech.gov.tw; 2 Division of Urology, Department of Surgery, Taipei City Hospital, Zhongxiao Branch, Taipei 115, Taiwan; E-Mail: shlu77777@gmail.com; 3 Department of Pharmacology, College of Medicine, Taipei Medical University, Taipei 110, Taiwan; E-Mail: tjaya_2002@yahoo.co.in; 4 Department of Cardiology, Cathay General Hospital, Taipei 106, Taiwan; E-Mail: change@seed.net.tw; 5 Department of Anatomy, Taipei Medical University, No. 250 Wu-Hsing Street, Taipei 110, Taiwan; E-Mail: thfong@tmu.edu.tw; 6 Department of Microbiology, Institute of Ophthalmology, Joseph Eye Hospital, Tiruchirappalli 620001, Tamil Nadu, India; E-Mail: thomasdiagnosticcentre@gmail.com; 7 Department of Emergency, Min-Sheng General Hospital, Taoyuan 330, Taiwan

**Keywords:** hinokitiol, melanoma, MMPs, antioxidant enzymes, histology, elastic fiber

## Abstract

Melanoma is extremely resistant to chemotherapy and the death rate is increasing hastily worldwide. Extracellular matrix promotes the migration and invasion of tumor cells through the production of matrix metalloproteinase (MMP)-2 and -9. Evidence has shown that natural dietary antioxidants are capable of inhibiting cancer cell growth. Our recent studies showed that hinokitiol, a natural bioactive compound, inhibited vascular smooth muscle cell proliferation and platelets aggregation. The present study is to investigate the anticancer efficacy of hinokitiol against B16-F10 melanoma cells via modulating tumor invasion factors MMPs, antioxidant enzymes *in vitro*. An *in vivo* mice model of histological investigation was performed to study the patterns of elastic and collagen fibers. Hinokitiol inhibited the expression and activity of MMPs-2 and -9 in B16-F10 melanoma cells, as measured by western blotting and gelatin zymography, respectively. An observed increase in protein expression of MMPs 2/9 in melanoma cells was significantly inhibited by hinokitiol. Notably, hinokitiol (1–5 μM) increased the activities of antioxidant enzymes catalase (CAT) and superoxide dismutase (SOD) from the reduction in melanoma cells. Also, hinokitiol (2–10 µM) concentration dependently reduced *in vitro* Fenton reaction induced hydroxyl radical (OH·) formation. An *in vivo* study showed that hinokitiol treatment increased elastic fibers (EF), collagens dispersion, and improved alveolar alterations in the lungs of B16/F10 injected mice. Overall, our findings propose that hinokitiol may be a potent anticancer candidate through down regulation of MMPs 9/2, reduction of OH· production and enhancement of antioxidant enzymes SOD and CAT.

## 1. Introduction

Metastatic melanoma is one of the utmost fatal and ambiguous cancers responsible for 4% of skin cancers and 75% of skin cancer-related deaths [1]. It can be controlled by multi-step processes such as tumor-induced angiogenesis, tumor invasion, and establishment of metastatic foci at the secondary site involving various molecules [2]. It has been proposed that activation of matrix metalloproteinase (MMP), a proteolytic enzyme in extracellular matrix (ECM), is diligently related with metastasis and cancer invasion. Among MMPs, MMPs-2, and -9 are predominantly convoluted in the metastasis process [3]. MMP-2 and MMP-9 are considered to be prognostic factors in many solid tumors, and also found to promote invasion and metastasis of malignant tumors [4]. Elevated levels of MMP-2 and MMP-9 are often correlated with malignancies of liver, brain, breast, prostate, cervical, ovarian, and other cancers. Studies have reported that MMPs-9 and -2 degrade native type IV collagen, denatured collagens (gelatin), and a number of types of native collagens [5]. They also reported to degrade elastic fibers [6]. Moreover, several lines of evidence suggested that cancer progression can be introverted by targeting antioxidant enzymes [7]. A previous study specifies that decrease in the activities of the antioxidant enzymes glutathione peroxidase (GPx), SOD, and CAT were found in lymphoma cell induced tumor in mice [8], and these enzymes are regarded as markers of malignant transformation [9]. Therefore, it has been recommended that natural antioxidant compounds can be used in various ways, either as cancer preventive agents or even as cancer therapy drugs [10]. 

In previous studies, some chemicals or peptides have reported to lower the invasive and metastatic ability of cancer cells by down regulating the activity and expression of MMPs [11]. Several novel MMP inhibitors are being developed and some have reached clinical trials as anti-metastatic or anti-cancer therapies [12]. On the other hand, studies showed that tumor metastasis is prevented by components such as l-carnosine, curcumin, and isorhamnetin via activation of antioxidants and inhibition of MMPs [13,14]. Despite numerous synthetic drugs being commercially obtainable to treat melanoma cancer, they produce severe probable side effects including nephrotoxicity, neurotoxicity, infertility, thromboembolic complications, hair loss, nausea, and myocardial infarction [15]. Therefore, it is essential to find more efficient natural products with less side effects for the prevention and abolition of melanoma tumors.

Hinokitiol, a natural tropolone-related compound isolated from the heartwood of *Chymacyparis taiwanensis*. It is a secondary metabolite, typical of plant cells, belonging to the phenol class. It is also known as as β-thujaplicin and has the chemical structure 2-hydroxy-4-isopropylcyclohepta-2,4,6-trien-1-one. Hiniokitiol has a wide variety of biochemical and pharmacological activities [16]. Since this compound owns several bioactivities such as antimicrobial [17], anti-bacterial [18], and neuroprotective activities [19], it has received increasing attention of several investigators. Our recent study shown that hinokitiol inhibits *in vitro* melanoma cell migration via inhibiting MMP-1 followed by suppressing NF-κB/MAPKs signaling pathways and *in vivo* tumor nodule formation [20]. A recent our study also shown hinokitiol inhibits VSMC proliferation via JNK1/2 and PLC-γ1 phosphorylation and limits the synthesis of specific cell cycle enzyme, PCNA [21]. With this background, in this study we aimed to evaluate the anticancer effects of hinokitiol against B16-F10 melanoma by assessing the status of tumor invasion factor-MMPs 2 and 9 along with the levels of intracellular antioxidant enzymes. Since tumor thickness is one of the important prognostic factors in patients with melanoma, an *in vivo* histological investigation was performed to study whether hinokitiol treatment alters the distribution of elastic fibers and collagens in the lungs of melanoma cells injected mice. 

## 2. Results and Discussion

### 2.1. Results

#### 2.1.1. Effects of Hinokitiol on MMP-2 and MMP-9 Expression 

**Figure 1:**
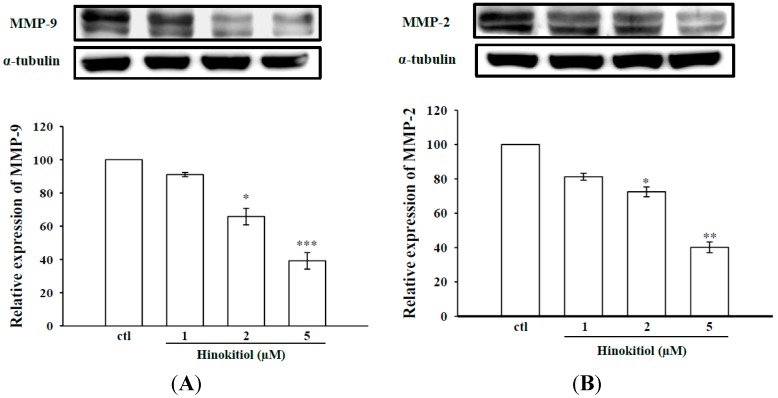
Effects of hinokitiol on MMPs-9 and -2 expression and in B16-F10 melanoma cells. B16-F10 melanoma cells were treated with different doses of hinokitiol (1–5 µM) in serum-free media for 24 h. Cell lysates were obtained to detect the MMP-9 (**A**) and MMP-2 (**B**) proteins expression using Western blotting. The figures are representative examples of three independent experiments. * Indicates the values are significantly different from the control (* *p* < 0.05, ** *p* < 0.01, *** *p* < 0.001).

Among other MMPs, MMPs-9 and -2 are known to play an important role in cancer cell invasion and metastasis, thus we examined whether hinokitiol could inhibit the expression of MMPs-9 and -2 in B16-F10 cells by using Western blot analysis. Figure 1A,B represents the protein expression levels of MMP-9 and MMP-2 in untreated and hinokitiol treated B16-F10 melanoma cells. The results showed an apparent increase in expression levels of MMPs-9 and -2 in untreated B16-F10 melanoma cells. This may due to increased concentration of ROS, which activates MMPs during stages of intense angiogenesis to disrupt ECM. However, when cells exposed with hinokitiol (1–5 µM), the intracellular protein levels of MMPs-9 and -2 were significantly reduced in a concentration-dependent manner.

#### 2.1.2. Effects of Hinokitiol on MMP-9 and MMP-2 Activities in B16-F10 Cells

**Figure 2:**
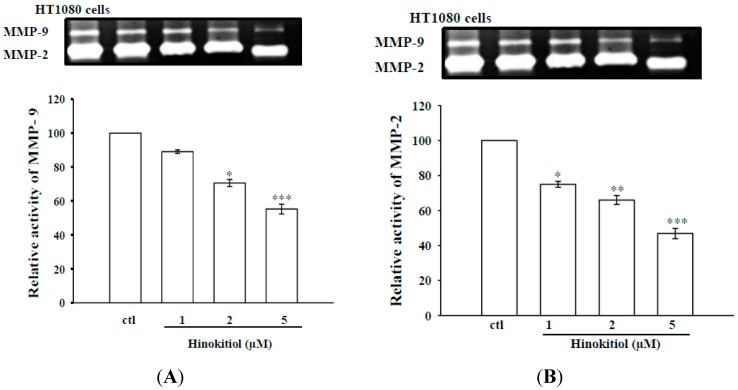
Effects of hinokitiol on MMPs-9 (**A**) and -2 (**B**) activity in B16-F10 melanoma cells. B16-F10 melanoma cells were treated with different doses of hinokitiol (1–5 µM) in serum-free media for 24 h. After treatment periods, media were collected for the assay of MMPs activity using gelatin zymography. The culture medium of HT1080 cells (the constitutive proMMP-2 and proMMP-9 were expressed) were used as control. The figures are representative examples of three independent experiments. * Indicates the values are significantly different from the control (* *p* < 0.05, ** *p* < 0.01, *** *p* < 0.001).

Activities of MMPs play an important role in tumor metastasis [22]. To clarify whether activities of MMPs are involved in anticancer activity by hinokitiol, we evaluated the effects of hinokitiol on MMP-9 and MMP-2 activities with the use of gelatin zymography. Data revealed that the gelatinolytic activities of MMPs-9 and -2 were constitutively activated in B16-F10 cells, whereas hinokitiol treatment significantly suppressed MMP-9 and MMP-2 enzymatic activities in a concentration-dependent manner (Figure 2A,B).

#### 2.1.3. Effects of Hinokitiol on Enzymatic Antioxidants CAT and SOD

**Figure 3:**
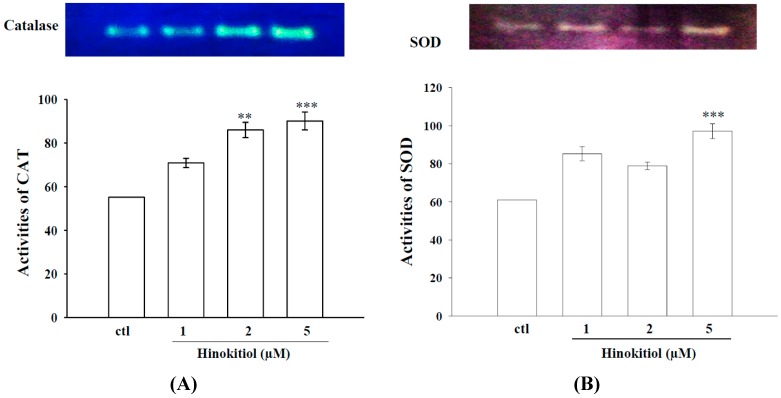
Effects of hinokitiol on CAT and SOD activity in B16-F10 melanoma cells. B16-F10 melanoma cells were treated with different doses of hinokitiol (1–5 µM) in serum-free media for 24 h. Cell lysates were obtained for the assay of CAT (**A**) and SOD (**B**) activities by using native-PAGE. The figures are representative examples of three independent experiments. * Indicates the values are significantly different from the control (** *p* < 0.01, *** *p* < 0.001).

Metastatic progression of cancer is associated with changes in antioxidant enzymes [23]. Figure 3A,B reveal the substrate gel activities of CAT and SOD in untreated and hinokitiol treated B16-F10 cells. The activities of the enzymes CAT and SOD were found to be decreased in untreated B16-F10 cells. However, a concentration-dependent increased activity of CAT was observed in hinokitiol exposed cells, the highest increase was detected in 5 µM hinokitiol treated cells. Similarly, the activity of SOD was also significantly increased at 5 µM hinokitiol treated cells whereas a modest increase in the SOD activity was only found in 1 and 2 µM hinokitiol treated cells. These results suggest that hinokitiol can destroy cancer cells through the up-regulation of antioxidant enzymes CAT and SOD.

#### 2.1.4. Hinokitiol Dampens OH· Production *in Vitro*

**Figure 4:**
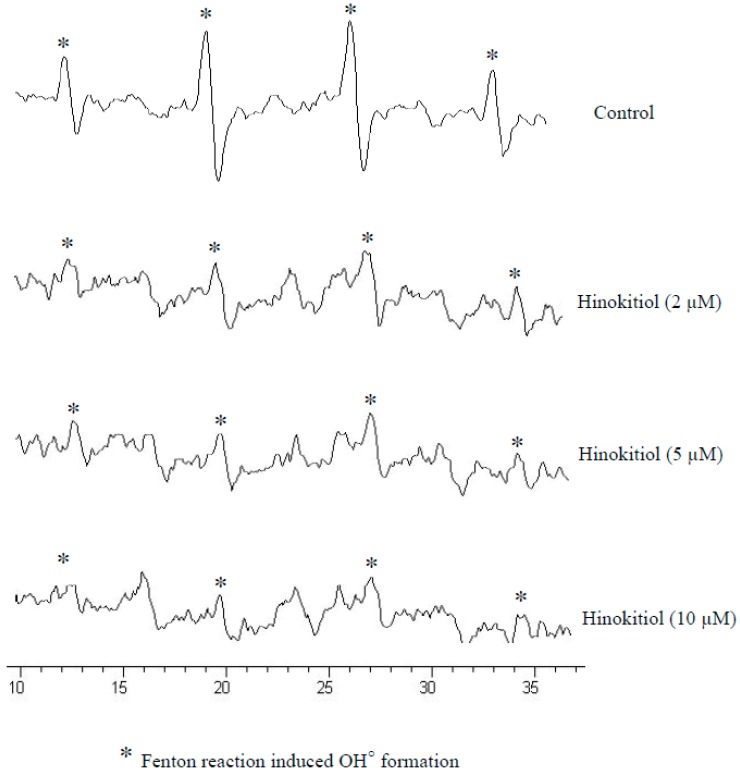
Effects of the extracts of hinokitiol on hydroxyl radical formation; ESR spectra shows the hinokitiol (2–10 µM) significantly inhibits Fenton reaction induced hydroxyl radical formation in a dose dependent manner. Data are presented as the mean ± S.E.M; * indicates Fenton reaction induced OH· formation.

Reactive oxygen species has been implicated in the pathogenesis of several chronic diseases including cancer [24]. DCFDA (non-fluorescent in a reduced state but fluorescent upon oxidation by ROS), cell-permeative ROS-sensitive dye was used to determine the scavenging ability of hinokitiol on Fenton reaction-induced OH· formation *in vitro*. Remarkably, hinokitiol markedly inhibited the Fenton reaction induced OH· formation in a concentration dependent manner as shown in Figure 4. Moreover, the maximum scavenging effect was found at 10 µM of hinokitiol administration. 

#### 2.1.5. Histopathological Observation in the Lungs of Hinokitiol Treated Mice

**Figure 5:**
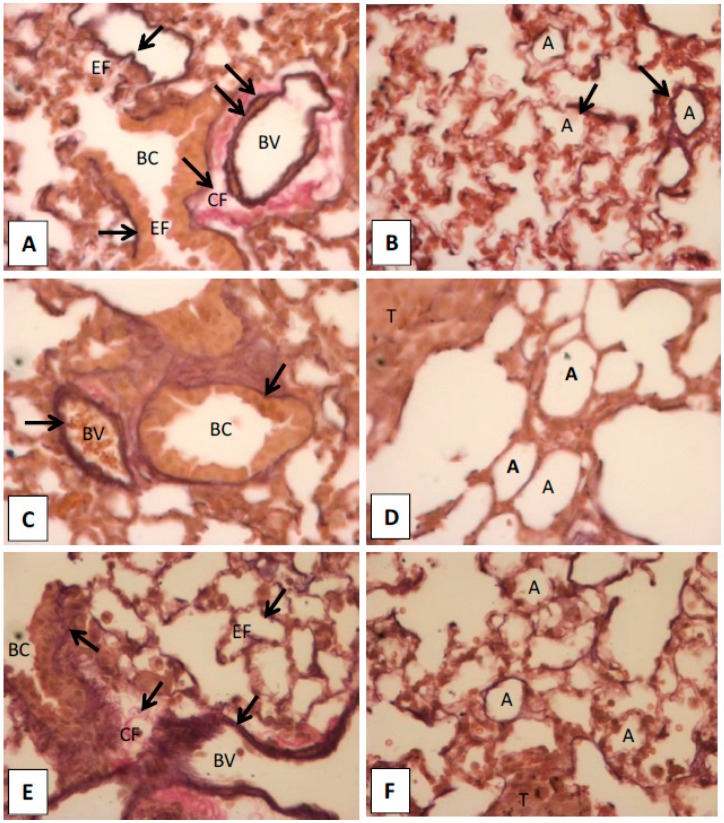
Elastic-van Gieson staining images of lungs. (**A**,**B**) are images of normal lung; (**C**,**D**) are images of lung with melanoma; (**E**,**F**) are images of cancer lung with hinokitiol-treatment. The elastic fibers (EF) are abundant in the inner and outer elastic lamina of blood vessels (BV), and boundary of bronchiole (BC). In normal and hinokitiol-treated groups, there are well defined alveoli and inter-alveolar septum. The EF and capillaries are rich in the interstitial of inter-alveolar septum. In addition, the collagen fibers (CF) are rich in adventitia of BV. In the tumor group, less EF was found around in the BV and BC, and enlarged alveoli occurred in the interstitial of the inter-alveolar compared with normal and hinokitiol-treatment groups. Bronchiole (BC), elastic fibers (EF), collagen fibers (CF), blood vessel (BV), alveoli (A), tumor (T).

The changed pattern of EF and collagens were suggested as diagnostic feature for certain types of cancers or as measure for identification of cancer progression, such as regression in melanoma [25,26]. Hence, we observed the EF and collagen patterns in the lungs of hinokitiol treated mice using EVG staining in histological analysis. We found abundant elastic fibers present in inner and outer elastic lamina of blood vessels (BV), and boundary of bronchiole (BC). Also, we observed well defined alveoli and inter-alveolar septum, and abundant EF and capillaries in the interstitial of inter-alveolar septum in normal and hinokitiol-treated groups. In addition, the collagen fibers (CF) are rich in adventitia of BV. However, very thin or low EF around in the BV and BC, and enlarged alveoli in the interstitial of inter-alveolar were found in the tumor group compared with normal and hinokitiol-treatment groups (Figure 5A–F). These results suggest that hinokitiol recovers melanoma induced degradation of EF and CF possibly through inactivation of MMPs9/2, because these enzymes are reported to degrade collagens [5] and elastic fibers [6].

### 2.2. Discussion

Our previous study demonstrated that hinokitiol inhibits MMP-1 by preventing nuclear factor kappa B (NF-κB) and cJUN activation, and by suppressing the mitogen activated protein kinase (MAPKs) signaling cascades, with consequence suppression of migration of B16-F10 melanoma cells [12]. In that study, we found that hinokitiol (1–5 μM) for 24 and 48 h incubation did not produce cytotoxic effect against melanoma cells. However, at the same incubation periods, hinokitiol showed a minimal cytotoxic effect when cells incubated at 10 μM. Based on these findings, in this present study, we have used hinokitiol at concentrations of 1, 2, and 5 μM. The results of the present study exhibit that hinokitiol possesses anticancer effects against melanoma through down regulation of MMPs-9 and -2, reduction of OH· radicals production and upregulation of antioxidants enzymes CAT and SOD. The most important finding of this study is that hinokitiol recovers B16-F10 cell mediated alteration of elastic fibers and collagen distribution (potential markers for clinical diagnosis of melanoma) in the lungs of C57/B6 mice.

MMPs are zinc dependent enzymes reported to involve in the initial stage of invasion and metastasis of various tumor cells [27]. Activation of MMPs has been observed in variety of metastatic tumor tissues, and hence numerous anticancer drug formulations mainly focus to attenuate the expression of MMPs. Inhibition of MMP expression and their activities are considered to be early targets for preventing cancer metastasis [28]. MMP-2 and MMP-9 are involved in the invasive metastatic potential of tumor cells [3,29]. Consistent with these events, here we found a marked increase in the expression of MMP-9 and MMP-2 in melanoma cells. In parallel zymography experiments, we confirmed this phenomenon that the activities of MMP-9 and MMP-2 were increased in melanoma cell. Nevertheless, in each case (western and zymography), hinokitiol exposure in melanoma cells significantly suppressed both expression and activities of MMPs-9 and -2. Our results demonstrated that anticancer effects of hinokitiol are associated with decreased MMPs. Consistent with this study, several previous investigations demonstrated that natural compounds such as quercetin [30], baicalein [31], and gallic acid [32] had anticancer activity through suppression of MMPs. 

It has been shown that the overexpression of SOD, which is dependent on the production of H_2_O_2_, increases the activity of transcription factors critical for MMP expression and also improves MMPs promoter activity. Also, the overexpression of SOD increases the mRNA levels of different MMPs, including MMPs-9 and -2, enhancing the metastatic capacity of fibrosarcoma cells implanted in immunodeficient mice [33]. A study has also been observed that the overexpression of SOD in the MCF-7 breast cancer cell line stimulates the activation of MMP-2 and increases the levels of ROS [34]. The activity of MMP-2 can be modulated according to intracellular levels of ROS. Radiation produces ROS, including superoxide anion (O2^−·^) and H_2_O_2_ and SOD transforms O2^−·^ into H_2_O_2_, which in turn activates MMP-2. The antioxidant enzymes that remove H_2_O_2_, such as CAT and GPx contribute to MMP-2 inactivation and reduce the tumor invasiveness derived from the action of this MMP [34]. Consistent with these studies, we tested whether hinikitiol inactivates MMPs-9 and -2 via scavenging free radicals and enhancing antioxidant enzymes CAT and SOD in melanoma cancer cells, and found that hinokitiol have the positive impact in these cells.

Scavenging of ROS by antioxidant enzymes may be an effective approach for inhibiting tumor metastasis [35], since various processes of tumor metastasis, such as adhesion, invasion and proliferation are associated with ROS generation [36]. Cells protect themselves from oxidative damage by the primary antioxidant enzymes include SOD, CAT, and GPx that play important roles in the elimination of free radicals. Concerning to these events, it was reported that curcumin had a potent antioxidant and suppressed tumor initiation, promotion, and metastasis [37]. l-carnosine has reported to have antimetastatic effect via increasing antioxidants and inhibiting the expression of MMP-9 in SK-Hep-1 cells [13]. Decrease in the activities of antioxidant enzymes SOD, CAT, and GPx in tumor induced rats have found to be increased when rats treated with an extract of *Ammania baccifera*, this effect may be due to the free radical quenching property of *A. baccifera* [9]. The antioxidant effects of the African medicinal plants *Moringa oleifera*, *Eremomastax speciose*, and *Aframomum melegueta* [38], and a vegetal product *Lavandula angustifolia* Miller essential oil [39] have thoroughly studied against UV-radiation induced B16-F10 melanoma cells. Corroborating with these outcomes, our results revealed that administration of hinokitiol significantly inactivates MMPs-9 and -2 via activating antioxidative enzymes SOD and CAT in melanoma cells and also inhibiting *in vitro* Fenton reaction-induced OH^·^ formation.

The changed pattern of elastic fibers has been observed in different types of cancers as a remarkable feature of the disease. The EF pattern is also suggested as a diagnostic feature for certain types of cancers, or as a measure for identification of cancer progression, such as regression in melanoma [26]. MMPs-9 and -2 enzymes degrade native type IV collagen, denatured collagens (gelatin), and several types of native collagens [5]. These enzymes also degrade elastic fibers [6]. Therefore in this study EVG staining is used to identify the pattern of EF and collagens, because these fibers located in the venous wall that will be stained dark violet and brown by this technique [40]. The decreased levels of elastic fibers were found in inner and outer elastic lamina of blood vessels, and boundary of bronchiole, reduced levels of collagen were observed in the lungs of melanoma injected mice. These results are consistent with a previous study that invasive malignant melanomas show markedly decreased absent elastic fibers within the nests of melanocytes and in the adjacent stroma [41]. Moreover, the patterns of EF and collagen were recovered almost into a near normal group when melanoma cells injected mice treated with hinokitiol. These results explain that hinokitiol recovered melanoma mediated EF and collagen in an *in vivo* mice model possibly resulting from the downregulation of MMPs-9 and -2 activation. 

## 3. Experimental Section

### 3.1. Materials

Hinikitiol (≥90%) was purchased from Sigma (St. Louis, MO, USA). Anti-mouse and anti-rabbit immunoglobulin G-conjugated horseradish peroxidase (HRP) was purchased from Amersham Biosciences (Sunnyvale, CA, USA) and/or Jackson-Immuno Research (West Grove, PA, USA). Sodium dodecylsulfate (SDS), phenylmethylsulfonyl fluoride (PMSF), leupeptin, aprotinin, sodium fluoride, sodium orthovanadate, sodium pyrophosphate, diethyl pyrocarbonate (DEPC), bovine serum albumin (BSA), potassium ferricyanide, ferric chloride, nitroblue tetrazolium (NBT), riboflavin were all purchased from Sigma-Aldrich (St. Louis, MO, USA). A mouse monoclonal antibody (m Ab) specific for human native 92-KDa MMP-9 was purchased from LabVision/NeoMarkers (Fremont, CA, USA). A rabbit polyclonal antibody (p Ab) specific for MMP-2 was purchased from Biovision (Milpitas, California, CA, USA). The Hybond-P polyvinylidene difluoride (PVDF) membrane, and enhanced chemiluminescence (ECL) Western blotting detection reagent and analysis system were obtained from Amersham (Buckinghamshire, UK). All other chemicals used in this study were of reagent grade. 

### 3.2. Cell Cultivation and Hinokitiol Administration

Mouse melanoma cells, B16-F10 was obtained from American Type Culture Collection (Manassas, VA, USA). Cells were cultured in RPMI1640 medium supplemented with l-glutamine (3.65 mM), penicillin (90 units/mL), streptomycin (90 μg/mL), HEPES (18 mM), NaHCO_3_ (23.57 mM), and 10% heat-inactivated fetal bovine serum (FBS) at 37 °C in humidified air with 5% CO_2_. In this study, B16-F10 cells were seeded at 5 × 10^4^ per well of Costar 6-well tissue culture plates in complete media until 90% confluent. After 24 h, cells were changed to serum-free media. Twenty-four hours after changing to serum-free media, cells were treated with hinokitiol (1–5 μM) for another 24 h. At the end of the incubation period, cell supernatants were collected and stored at −80 °C for the Western blot assay.

### 3.3. Western Blot Analysis of MMP-9 and MMP-2

B16-F10 cells (5 × 10^4^ cells/well) were seeded in 6-well plates and grown to 80%–90% confluence. Confluence cells were pre-incubated with 1–5 µM hinokitiol for 24 h. After the experimental periods, the proteins were extracted with 60 µL lysis buffer (20 mM/L Tris-HCl, pH 7.5; 1 mM/L MgCl_2_; 125 mM/L NaCl; 1% Triton X-100; 1 mM/L phenylmethylsulfonyl fluoride; 10 µg/mL leupeptin and 10 µg/mL aprotinin). The lysates were centrifuged at 12,000× *g* for 20 min at 4 °C. Samples containing 50 µg of protein were separated by a 10% sodium dodecylsulfate polyacrylamide gel electrophoresis (SDS-PAGE), and the proteins were electrotransferred on to a PVDF membrane using a Bio-Rad semi dry transfer unit (Hercules, CA, USA). For immunodetection, the PVDF membrane was blocked in 5% nonfat dry milk in Tris-buffered saline with TBST (10 mM Tris-base, 100 mM NaCl, and 0.01% Tween 20) for 40 min at room temperature, and then probed with MMP-9 and MMP-2 antibodies at a dilution of 1:1000 for 2 h at room temperature. The blots were then extensively washed in TBST and then incubated with HRP-linked anti-mouse IgG or anti-rabbit IgG (diluted 1:3000 in TBST) for 1 h. The immunoreactive bands were visualized with enhanced chemiluminescent reagents (ECL, Amersham). Quantitative data were obtained using a computing densitometer equipped with a scientific imaging system (Biospectrum AC System, UVP).

### 3.4. Gelatin Zymographic Assay for MMP-9 and MMP-2

Activities of MMP-9 and MMP-2 were measured by gelatin zymography, an extremely sensitive technique that can detect picogram quantities as described by our previous study [42]. To this, B16-F10 cells (5 × 10^4^ cells/well) were seeded on 6-well plates and grown to 90% confluence in 2 mL of RPMI1640 growth medium. The cells were maintained in serum-free media and then treated with various concentrations of hinokitiol for 24 h. Equal amounts of conditioned media were mixed with non-reducing buffer (500 mM Tris-HCl, 25% glycerol, 10% SDS, and 0.32% bromophenol blue; pH 6.8), incubated for 5 min on ice and electrophoresed onto 10% polyacrylamide gels containing 1 mg/mL gelatin. After electrophoresis, the gels were washed twice with 2.5% Triton X-100 to remove the SDS and then incubated with reacting buffer containing 50 mM Tris-base, 200 mM NaCl, 5 mM CaCl_2_, and 0.02% Brij 35 (pH 7.5) for 17 h in a closed container at 37 °C. At the end of the incubation, the gels were fixed with a fixing solution (7% acetic acid and 40% methanol, *v*/*v*) for 30 min. Gels were stained with a solution of Colloidal Brilliant Blue G in 27% methanol for 30 min. Finally, a destaining solution (10% acetic acid in 25% methanol) was used to adjust the clear conditions. Clear zones (bands) against the blue background indicated the presence of degradative activity of MMPs-9 and -2.

### 3.5. Non-Denaturing Polyacrylamide Gel Electrophoresis (Native-PAGE) for CAT and SOD Activity Assay

To further confirm the enzymatic activity assay of CAT and SOD, native-PAGE was carried out except that SDS was omitted from all buffers and the samples were not boiled before electrophoresis. The enzymes were run on the basis of equal amounts of protein (50 µg) in an 8% gel for CAT and 10% gel for SOD. Electrophoretic separation was performed at 4 °C with a constant power supply of 80 V for stacking gel and 100 V for separating gel. Staining for the activity of each enzyme was performed separately as follows:

(i) CAT activity was detected by the method of Woodbury *et al.* [43]. Here, the gel was soaked in 5 mM H_2_O_2_ solution for 10 min and then washed with water and stained with a reaction mixture containing 1% potassium ferricyanide (*w*/*v*) and 1% ferric chloride. The enzyme appeared as a yellow band superimposed on a dark green background. The reaction was terminated by adding water and the gel was photographed at once.
(ii) SOD activity was identified by the method of Beauchamp and Fridovich [44]. The gel was soaked in 50 mM Tris-HCl buffer (pH 8) containing 10 mg nitroblue tetrazolium (NBT), 1 mg ethylene diamine tetra acetic acid (EDTA), and 2 mg riboflavin (50 mL final volume), and kept in the dark for 30 min. The gel was then placed on an illuminated light box to locate the area of SOD activity, which appeared as a clear zone on a bluish-violet background.

### 3.6. Detection of Hydroxyl Radical (HO^·^) Formation Using Electron Spin Resonance (ESR) Spectrometry

The ESR method used a Bruker EMX ESR spectrometer (Billerica, MA, USA) as described previously [45]. In brief, a Fenton reaction solution (50 µM FeSO_4_ + 2 mM H_2_O_2_) was pretreated with a solvent control (0.1% DMSO) or hinokitiol (2, 5 and 10 µM) for 10 min. The rate of hydroxyl radical-scavenging activity was defined by the following equation: inhibition rate = 1 − [signal height (hinokitiol)/signal height (solvent control)].

### 3.7. In Vivo Histological Analysis of Lungs to Detect the Elastic Fibers and Collagens

Despite hematoxylin-eosin (H & E) stained histopathology is believed to be the gold standard for diagnosing different types of lung cancer in clinics, it becomes difficult sometimes to differentiate the pattern of elastic fibers and collagen in the regressed areas of melanoma. Thus we used elastic van Gieson (EVG) stain to analyze the distribution of the elastic fibers and collagen in the lungs of hinokitiol treated melanoma cell-injected mice. Briefly, B16-F10 melanoma cells were harvested with trypsin solution and resuspended to appropriate concentrations in PBS and injected into the lateral tail vein of C57BL/6 mice. Mice were randomly divided into three groups of 10 mice each: Gr-I: normal control group; G-II: B16-F10 cells (5 × 10^4^ cells/ mouse) injected group; G-III: One hour after injecting of B16-F10 melanoma tumor cells, mice were intraperitoneally treated with a single dose of hinokitio 3 (20 µg/mouse). Fourteen days later, the mice were killed by cervical dislocation; the lungs were removed, washed with PBS, and fixed with 10% formaldehyde, dehydrated, and embedded in paraffin wax for histological studies. From the blocks, 5 μm sections were then stained with EVG, mounted in DPX and examined under a microscope for histopathological changes in lungs as described before [46]. 

### 3.8. Statistical Analysis

The experimental results are expressed as the mean ± SEM and are accompanied by the number of observations. For analysis of the results, a one-way analysis of variance (ANOVA) test was performed using the Sigma Stat v3.5 software. When group comparisons showed a significant difference, the Student-Newman-Keuls test was used. *p*-Value less than 0.05 were considered statistically significant.

## 4. Conclusions

Overall, our results demonstrated that hinokitiol exerted anticancer effects via downregulation of proteolytic enzymes MMP-9 and -2, enhancement of antioxidative enzymes SOD and CAT, and also inhibiting OH· radical formation. Hinokitiol also restored melanoma induced degradation of elastic fibers and collagen in the lungs of metastatic bearing mice. These results suggest that hinokitiol has potent anticancer activity *in vitro* via modulation of MMPs-9 and -2 and enzymatic antioxidant system and by recovering of EF and collagen *in vivo*. 
